# Supplementary material for: Treatment effects beyond the mean using distributional regression: Methods and guidance
Source: PLoS One. 2020 Feb 14;15(2):e0226514. doi: 10.1371/journal.pone.0226514 (PMC7021287; doi:10.1371/journal.pone.0226514)
Supplement: Attachment — Submitted filename: response_to_reviewers_gamlss_in_ie.pdf [file pone.0226514.s005.pdf]

Response to reviewers

November 5, 2019

We thank both reviewers for the comments that helped us in improving upon the original submission. The most important changes relate to

- a more detailed discussion of the impact of spatial autorcorrelation and the ability of GAMLSS to handle such spatial effects.
- shortening and streamlining the presentation at various places to make the paper more easily accessible.
- explaining in more detail for which kind of data and situations GAMLSS provide specific potential.

Reviewer 1

- *This paper proposes the GAMLSS for analyzing treatment effects beyond the mean. The paper is well written and easily accessible. However, it is somewhat lengthy.*

  Thank you very much for your positive assessment of our paper. Following your suggestion, we went through the complete paper in detail to identify areas where the presentation could be condensed.

- *Moreover, as indicated in the Introduction section, the GAMLSS has already been advocated by Rigby and Stasinopoulos (2005). The authors should further highlight their academic contributions of this research: The first work on using the GAMLSS for analyzing treatment effects beyond the mean?*

  The contribution of the paper is introducing GAMLSS to treatment effect analyses beyond the mean. The paper also intends to provide a hands-on guidance for applied researchers on how to conduct such an analysis. We now highlight this contribution more clearly in the introduction and also streamlined the presentation at various places to emphasize the contribution throughout the article.

- *The authors suggested that the proposed GAMLSS is able to account for spatial effects/heterogeneities in the response variable. Spatial autocorrelation is a typical kind of spatial effects, which has been found in many*

real-world observation data. Accounting for the spatial autocorrelation in the statistical models would help improve model estimation, reduce model misspecification, and avoid misidentification of significant factors. The following is some examples in the field of traffic safety analysis:

- *Zeng Q., Gu W., Zhang X., Wen H., Lee J., Hao W. (2019). Analyzing freeway crash severity using a Bayesian spatial generalized ordered logit model with conditional autoregressive priors. Accident Analysis & Prevention, 127, 87-95.*
- *Zeng, Q., Guo, Q., Wong, S. C., Wen, H., Huang, H., Pei, X. (2019). Jointly modeling area-level crash rates by severity: A Bayesian multivariate random-parameters spatio-temporal Tobit regression. Transportmetrica A: Transport Science, 15 (2): 1867-1884.*
- *Zeng, Q., Wen, H., Huang, H., Abdel-Aty, M. (2017). A Bayesian spatial random parameters Tobit model for analyzing crash rates on roadway segments. Accident Analysis & Prevention, 100, 37-43.*

In the existence of spatial autocorrelation, the response variables become correlated. Is the GAMLSS able to handle this issue? At least, the general framework illustrated in Section 2.1 did not show this ability.

We completely agree that accounting for spatial autocorrelation is a common and important challenge in statistical analyses and hence follow your suggestion to discuss this point in more detail in the paper. GAMLSS are readily able to include different types of spatial effects. These spatial effects include stationary Gaussian random fields based on various types of covariance functions when spatial information is available in terms of continuous coordinates of observations. In case of areal data where instead of exact coordinates only information on the regional allocation of observations is available, Gaussian Markov random fields can be used. We now elaborate on this aspect and the benefits of adjusting for spatial effects in more detail in Section 2.2 of the paper. We also included additional references on this aspect including some of your suggestions.

- *In line 273, some references should be cited on the tobit model, such as:*

  - *Zeng, Q., Wen, H., Huang, H., Abdel-Aty, M. (2017). A Bayesian spatial random parameters Tobit model for analyzing crash rates on roadway segments. Accident Analysis & Prevention, 100, 37-43.*
  - *Zeng Q., Wen H., Huang H., Pei X., Wong S.C. (2018). Incorporating temporal correlation into a multivariate random parameters Tobit model for modeling crash rate by injury severity. Transportmetrica A: Transport Science, 14 (3): 177-191.*

  Following your suggestion, we included the paper by Zeng et al (2017) as a reference for the tobit model.

- *In line 154, Equation (3) should be Equation (4).*

  Fixed.

Reviewer 2

- *This paper is a little bit lengthy, so it is not easy to follow. One suggestion is that the comparison of the GAMLSS and previous models could be put together; therefore the readers can easily know the contribution of this paper.*

  Following your suggestion and a similar request from the other referee, we shortened and streamlined the presentation of the paper at various places to make it more easily accessible.

- *As the authors stated, there are several advantages of GAMLSS; for example, it can consider panel data, random effect, discrete and multivariate distributions, and over-dispersion and zero-inflation. There need be some references for the reasons why this consideration is important. For example, the following ones about panel data [1-2], discrete and multivariate distributions [3-4], over-dispersion and zero-inflation [2, 5]*

  [1] Analysis of hourly crash likelihood using unbalanced panel data mixed logit model and real-time driving environmental big data. JOURNAL OF SAFETY RESEARCH. 2018, 65: 153-159.

  [2] Crash Frequency Modeling Using Real-Time Environmental and Traffic Data and Unbalanced Panel Data Models, International Journal of Environmental Research and Public Health, 2016, 13(6), 609.

  [3] Injury severities of truck drivers in single- and multi-vehicle accidents on rural highway, Accident Analysis and Prevention, 2011, 43(5), 1677-1688.

  [4] Investigation on the Injury Severity of Drivers in Rear-End Collisions Between Cars Using a Random Parameters Bivariate Ordered Probit Model, International Journal of Environmental Research and Public Health, 2019, 16(14) , 2632.

  [5] Crash Frequency Analysis Using Hurdle Models with Random Effects Considering Short-Term Panel Data", International Journal of Environmental Research and Public Health, 2016, 13(11) ,1043.

  Thank you for pointing us at these references to emphasize why considering panel data, random effect, discrete and multivariate distributions, over-dispersion and zero-inflation is important. We followed your suggestion and have now added more details and some of your references concerning distributions and their applications in Section 2.1 and modelling abilities in terms of the regression predictor in Section 2.2.

- *For the model estimation methods, maximum likelihood and Bayesian methods, the possible reference [1-5] maybe also be referred.*

  We also provide additional references on likelihood and Bayesian inference for GAMLSS at the end of Section 2.2. We restricted our references to those that discuss details on inference in GAMLSS-type regression models

and therefore refrained from including the suggested references at this point.

- *"Figure 3" could be "Fig. 3"*

  Fixed.

- *For the marginal effects, the authors need to mention which kind of calculation is used, for example, elasticity and so on, which could be referred to [3].*

  We refer to marginal (treatment) effects defined by a change in features in the outcome distribution when changing the binary (treatment) variable from 0 to 1 while fixing all other explanatory variables at specified values. The latter are usually mean values for continuous variables and modes for categorical variables. We clarified this in the manuscript, see the paragraph on "Reporting and interpreting the results" in Section 4.2.
